# Cancer Stem Cells Are Possible Key Players in Regulating Anti-Tumor Immune Responses: The Role of Immunomodulating Molecules and MicroRNAs

**DOI:** 10.3390/cancers13071674

**Published:** 2021-04-02

**Authors:** Sara Tomei, Ola Ibnaof, Shilpa Ravindran, Soldano Ferrone, Cristina Maccalli

**Affiliations:** 1 Research Department, Sidra Medicine, Doha, Qatar; stomei@sidra.org (S.T.); Ibnouf-c@sidra.org (O.I.); ravindranshilpa@gmail.com (S.R.); 2 Department of Surgery, Massachusetts General Hospital, Harvard Medical School, Boston, MA 02115, USA; sferrone@mgh.harvard.edu

**Keywords:** cancer stem cells/cancer initiating cells, tumor microenvironment, immune responses, immunomodulating molecules, microRNAs

## Abstract

**Simple Summary:**

This review provides a critical overview of the state of the art of the characterization of the immunological profile of a rare component of the tumors, denominated cancer stem cells (CSCs) or cancer initiating cells (CICs). These cells are endowed with the ability to form and propagate tumors and resistance to therapies, including the most innovative approaches. These investigations contribute to understanding the mechanisms regulating the interaction of CSCs/CICs with the immune system and identifying novel therapeutic approaches to render these cells visible and susceptible to immune responses.

**Abstract:**

Cancer cells endowed with stemness properties and representing a rare population of cells within malignant lesions have been isolated from tumors with different histological origins. These cells, denominated as cancer stem cells (CSCs) or cancer initiating cells (CICs), are responsible for tumor initiation, progression and resistance to therapies, including immunotherapy. The dynamic crosstalk of CSCs/CICs with the tumor microenvironment orchestrates their fate and plasticity as well as their immunogenicity. CSCs/CICs, as observed in multiple studies, display either the aberrant expression of immunomodulatory molecules or suboptimal levels of molecules involved in antigen processing and presentation, leading to immune evasion. MicroRNAs (miRNAs) that can regulate either stemness properties or their immunological profile, with in some cases dual functions, can provide insights into these mechanisms and possible interventions to develop novel therapeutic strategies targeting CSCs/CICs and reverting their immunogenicity. In this review, we provide an overview of the immunoregulatory features of CSCs/CICs including miRNA profiles involved in the regulation of the interplay between stemness and immunological properties.

## 1. Introduction

Tumors are composed of multiple and heterogeneous sub-populations of cells including those endowed with self-renewal and multipotency features, denominated as cancer stem cells (CSCs) or cancer initiating cells (CICs) [1,2,3,4,5,6,7]. Bonnet et al. reported for the first time the presence of CSCs/CICs in acute myeloid leukemia [8]. Later, cells endowed with stemness properties were isolated from solid tumors, such as brain, breast, colorectal (CRC) and ovarian cancer [9,10,11,12]. Various methods are used for the identification of CSCs/CICs, including the analysis of expression of markers using flow cytometry, the detection of side population by Hoechst method, the tumor sphere formation, the aldehyde dehydrogenase-1 (ALDH-1) activity assay and the tumorigenicity in in vivo immunodeficient mice [10,13,14,15]. Molecules such as sex determining region-Y-box 2 (SOX-2), octamer binding transcription factor (OCT) 3/4, ALDH-1, leucine-rich repeat-containing G-protein-coupled receptor 5 (LGR5), Kruppel-like factors 4 (KLF4), NANOG, ATP-binding cassette, sub-family A (ABC1), member 5 (ABCA5), CD133, CD44, CD24 and their differential expression patterns are often used to examine the stemness properties [16]. Nevertheless, CSC/CIC-specific and unique identifier markers are not available. Moreover, models that can resemble the in vivo tumorigenesis in the presence of the tumor microenvironment (TME) have not been developed, leading to several difficulties in identifying unique and standardized methods to study CSCs/CICs [3,7,17,18,19,20,21,22,23].

The CSCs/CICs play a critical role in the cancer initiation, maintenance, recurrence and progression and in the resistance to standard therapies [1,2,12,24,25,26,27,28]. The ability to cycle between proliferation and quiescence, the upregulation of DNA damage repair mechanisms, the regulation by epigenetic mechanisms of survival and proliferation and the expression of ATP-binding cassette (ABC) drug pumps are among the principal molecular pathways aberrantly expressed in CSCs/CICs and that affect the resistance of these cells to chemotherapy and radiotherapy [9,29,30]. Epithelial-to-mesenchymal transition (EMT), a process that transforms epithelial cells into mesenchymal-like cells, which causes disruption of cell-to-cell adherence, loss of apical-basal polarity, matrix remodeling, increased motility and invasiveness, promotes the development of CSCs/CICs [31,32,33,34,35,36,37,38]. This process is also orchestrated by the inflammation and the tumor microenvironment (TME) [32,33,35,39,40,41,42,43]. CSCs/CICs, similarly to normal stem cells, reside in a “niche”, represented by the TME, which consists of fibroblasts, endothelial, stromal, mesenchymal and immune cells and extracellular matrix (ECM) [4,44]. The niche is required for their survival, cycling from quiescence to proliferation, maintenance and regulation of stemness properties [45]. The interaction of CSCs/CICs with the niche is also responsible of their high grade of heterogeneity and plasticity [46,47,48,49,50]. The crosstalk of CSCs/CICs with the niche/TME, can influence the fate of CSCs as well as their resistance to therapies [51,52]. CSCs/CICs have been shown also to be resistant to immune-based interventions [53,54,55]. The suboptimal immunogenicity of CSCs/CICs is a hallmark of these cells, which, similarly to normal stem cells, determines the immune privileged make up of these cells [16,49,55,56,57,58,59,60,61]. The mechanisms leading to the immunomodulating properties of CSCs/CICs are mostly unknown, therefore gaining a more comprehensive characterization of the genomic and immunological profile of these cells will contribute to dissect their regulatory pathways. Few immunomodulatory features associated with CSCs/CICs have been reported, such as their ability either to express on the surface of the cells or to secrete immunosuppressive cytokines/factors (e.g., IL-4, IL-10, IL-13, immune checkpoints, Galectin-3, CXCL12, TGF-β and CD200) [53,55,61,62,63,64,65]. These factors drive the differentiation of immune cells to negative regulatory subtypes, such as T regulatory cells (Tregs), myeloid-derived suppressor cells (MDSCs), immature dendritic cells (iDCs) with tolerogenic properties and macrophages. As a consequence, the anti-tumor reactivity of cytotoxic T cells and NK cells are inhibited. Moreover, suboptimal levels of MHC molecules, antigen processing machinery (APM) and tumor associated antigens (TAAs) have been reported by several groups, leading to an inefficient T cell-mediated recognition and killing of CSCs/CICs [53,55,61,62,66].

Indoleamine 2,3-dioxygenase (IDO) is a monomeric oxidoreductase that catalyzes the first and rate-limiting step in tryptophan degradation, leading to subsequent production of the bioactive tryptophan metabolites kynurenine [67,68]. IDO-mediated depletion of tryptophan can modulate the immune system to arrest the inflammation, suppress the immunity to cancer and inhibit the autoimmunity. IDO is over-expressed by a variety of solid tumors, such as breast and prostate cancer [67,68,69]. IDO has been identified as a critical micro-environmental factor involved in aiding immune escape to an immunosuppressive TME. This enzyme can also play a role in orchestrating the immunosuppressive properties of CSCs/CICs and in their interaction with the TME [55,70].

MicroRNAs (miRNAs) are non-coding RNAs that bind to specific target mRNA(s) in order to regulate the expression of the gene(s) [71]. MiRNAs can also regulate the immune functions of cells [72,73,74,75,76,77]. In this line, they might play a relevant role in the immunological make up of CSCs/CICs. However, the regulatory mechanisms of miRNAs in the immunological functions of CSCs/CICs have not been fully clarified. In this review, we provide an overview of the immunomodulatory activities of CSCs/CICs with special focus on the molecular profiling of molecules with immunoregulatory functions and microRNAs.

## 2. Mechanisms of Immune Resistance of CSCs/CICs to Cell Mediated Immune Responses

The ability of CSCs/CICs to elicit T cell-mediated responses is dependent on the efficiency in expressing HLA and APM molecules [53,55,78]. Various studies on CSCs/CICs from colorectal cancer (CRC), melanoma and glioblastoma multiforme (GBM) reported a defective expression of HLA class I and antigen processing machinery (APM) molecules [53,65,66,79,80]. Proteins through ubiquitination enter into the degradation process mediated by proteasome/immunoproteasome molecules (low-molecular-weight proteins, LMPs; LMP2, LMP7 and LMP10), resulting in the generation of antigenic peptides with elevated affinity for HLA class I molecules [81,82]. Then, the transporters of antigen processing (TAP1 and TAP2) mobilize the antigenic peptides to the endoplasmic reticulum, where peptides are loaded onto HLA class I heavy chains with the aid of chaperone molecules, calnexin, calreticulin, ERp57 and tapasin [81,82]. This leads to the assembly of the trimeric complex, which consists of HLA class I heavy chain, β2-microglobulin and the antigenic peptide. These complexes will shuttle to the cell membrane to be exposed extracellularly for T cell receptor (TCR) recognition by T cells [81,82]. Failure along different steps of this process will result in impaired T-cell mediated immune responses. CSCs/CICs isolated from solid tumors have shown defective/suboptimal expression of HLA class I and APM molecules; these aberrant expressions have been linked to their evasion from T cell recognition [55,56,57]. However, there are limited data on its mechanistic cause. CSCs/CICs have been shown to be resistant to modulating agents that can upregulate the expression of these molecules, such as IFN (α/γ) and the demethylating agent (5-Aza CdR) [53,61,80]. Similar results were reported in GBM, where the expression of HLA class I was absent in CD133^+^ GBM CSCs/CICs [66]. In the case of ABCB5^+^ melanoma initiating cells, the expression of HLA molecules was shown to be lower as compared with the ABCB5^−^ melanoma non-stem cells [79]. Busse et al. demonstrated that, even though similar levels of mRNA for HLA and APM molecules were detected in CSCs/CICs and bulk tumor cells, the protein levels were downregulated in cells with stemness properties, suggesting that post-transcriptional mechanisms can lead to suboptimal antigen processing and presentation in these cells [83]. This evidence correlates with the data showing that CSCs/CICs failed in eliciting tumor-specific T cell-mediated responses [61]. On the other hand, NK cells can efficiently recognize and lyse CSCs/CICs when they are defective of the expression of HLA class I molecules and display detectable levels of the ligands of NK activatory receptors [84,85,86,87]. 

The stress-inducible major histocompatibility complex class I chain A related (MICA/B) and UL16 binding protein (ULBPs) represent the ligands of the natural killer group 2 member D (NKG2D) receptor, that is either an activatory or inhibitory receptor for NK and T cells, respectively [88,89,90]. The expression of these ligands has been shown to correlate with the prognosis of solid tumors and patients’ clinical outcome [88,89,90,91,92]. Suboptimal or negative levels of the expression of these ligands were detected in CSCs/CICs from GBM, affecting the efficiency of these cells in eliciting tumor-specific immune responses [55,56,61]. On the other hand, the detection of these molecules on the surface of CRC-CSCs/CICs together with low expression of HLA molecules was sufficient to sustain their susceptibility to NK cell reactivity [55,80,85]. 

In line with the above observations, the expression of TAAs in CSCs/CICs has been explored as possible tool for immunotherapy interventions [16,55,93]. TAAs can be grouped as the following: (*A*) Overexpressed antigens are generally expressed at very high levels in tumor cells, but usually detected at lower levels in normal tissues. Overexpressed TAAs can be sub-categorized into: (*A.1*) Antigens expressed in ubiquitous normal organs such as the proto-oncogene human epidermal growth factor receptor type 2 (HER-2) that are overexpressed in glioma stem cells [94]. In breast cancer stem cells (BCSCs), HER-2 is an important regulator, and its blockade in breast cancer cell line reduces the CSCs/CICs population [95]. The human telomerase reverse transcriptase (hTERT) is overexpressed in CD44^+^ BCSCs [95]. In colon CSCs/CICs, the centrosomal protein 55 (CEP55) is highly expressed [96]. Additionally, apoptotic inhibitory proteins have been reported in CSCs/CICs such as Survivin and B cell lymphoma 2 (Bcl-2) [94,96]. (*A.2*) Stem cell-related antigens. These TAAs, such as SOX-2, OCT 3/4 and ALDH-1 that are overexpressed in CSCs, have a pivotal role in tumorigenesis and are expressed at lower levels in normal stem cells. However, the efficacy of targeting these types of TAAs is limited by the pre-existence in the host of tolerant T cells directed to self-antigens [93,94,96,97]. (*B*) Cancer testis (CT) antigens, such as Melanoma Antigens (MAGE, B melanoma antigen (BAGE), G antigen 1 (GAGE), X antigen 1 (XAGE1), sperm protein associated with the nucleus on the chromosome X (SPANX), New York esophageal squamous cell carcinoma-1 (NY-ESO-1), etc., are expressed on multiple type of tumors [94]. The expression of these antigens in normal tissues is limited to germ cells, and, due to disruption in gene regulation, in malignant cells, they become deregulated and targets of T cells [98]. CT antigens exert oncological functions by enhancing tumor proliferation and metastasis, which eventually increase the resistance to treatment [98,99]. It has been reported that certain novel CT antigens, such as the olfactory receptor family 7 subfamily C member (OLF7C1), are preferentially expressed in CRC-CSCs/CICs, in association with poor prognosis of patients [94,98,99]. (*C*) Neoantigens or mutated antigens derived from somatic point mutations in cancer cells (e.g., multiple myeloma-1 (MUM-1), cyclin dependent kinase 4 (CDK4), meiotic spindle formation protein 1 (ME1), actinin alpha 4 (ACTN4), HLA-A2 and mothers against decapentaplegic homolog 4 (*SMAD4*)) [100,101,102,103] are a category comprised of high immunogenic TAAs, since they are not expressed in normal cells and, thus tolerogenic mechanism do not occur. Of note, the somatic mutation in *SMAD4* gene expressed by CRC-CSCs/CICs could induce tumor-specific T cells responses [103]. Nevertheless, controversial data of the potential targeting of differentiated, self- and CT-TAAs in CSCs/CICs have been reported [2,56]. 

In summary, the suboptimal expression of HLA and antigen processing machinery, together with the lack of information for CSC/CIC-specific TAAs, render these cells less susceptible to cell-mediated immune responses and also less likely to be efficiently targeted by immunotherapy approaches. The lack of in vivo animal models that might resemble the crosstalk of CSCs/CICs with TME represents a limitation in the investigation of the mechanisms leading to the low immunogenic profile of these cells. Nevertheless, the lack of standardization of the methods for the isolation in vitro and the biological characterization of CSCs/CICs and their high grade of plasticity represent the principal limitations to obtain definitive results in terms of their immunogenicity. Therefore, investigations to further gain knowledge in the profile of TAAs and in the mechanisms regulating the immunological properties of stem tumor cells are needed. 

## 3. Interaction of Immune Cells with CSCs/CICs 

**Figure 1:**
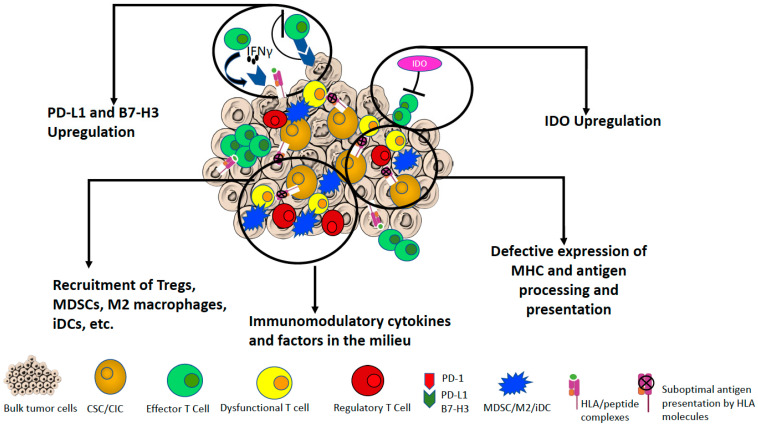
Principal mechanisms of immune evasion by CSCs/CICs. The immunomodulatory mechanisms by CSCs/CICs and the crosstalk with the TME that lead to impaired T cell-mediated responses are reviewed. The aberrant expression of multiple signaling pathways leads to the inefficient recognition and attack of CSCs/CICs by immune system. Among these pathways are: (1) the suboptimal expression of HLA molecules and APM, as well as of co-stimulatory molecules that cause the inability of T cells to recognize and kill the CSCs/CICs and their inefficient stimulation leading to their anergic/dysfunctional status; (2) the upregulation of the immune checkpoints, such as PD-1/PD-L1 and B7-H3 and the tryptophan catabolism by IDO that lead to impairment of effector T cell responses and the differentiation of immune suppressive immune cells (Tregs) and dysfunctional T lymphocytes); (3) the expression of pro-inflammatory cytokines (e.g., IL-6, IL-8, IL-10 and IL-13) and chemokines that drives the differentiation of immune cells toward suppressive subtypes (e.g., Tregs, M2 macrophages, MDSCs and iDCS); and (4) IFN-γ can play a dual effect in mediating both the anti-tumor effector functions and in up-modulating the negative regulator IDO. Moreover, TGFβ-1 in the TME can also regulate the expression of IDO. APM, antigen processing machinery; CSC/CIC, cancer stem cell/cancer initiating cell; HLA, human leukocyte antigen; iDC: immature and tolerogenic dendritic cells; IDO, Indoleamine 2,3- dioxygenase; IFN, interferon; IL-4, Interleukin 4; IL-6, Interleukin 6; IL-10, Interleukin 10; IL-13, Interleukin 13; MDSC, myeloid derived suppressor cell; M2 macrophages: immunomodulatory/suppressive macrophages; PD-1, programmed cell death 1; PD-L1, programmed cell death ligand 1; TME, tumor microenvironment; Treg, T regulatory cells.

The growth of tumor cells can be initially controlled by both innate and adaptive immune responses, followed by the equilibrium status in which tumor cells through the acquisition of further mutations and the development of immunomodulatory features, acquire an immunological dormancy. The final phase is represented by the escape of cancer cells from immune surveillance [104,105,106]. This theory has been defined as the 3E (Elimination, Equilibrium, Escape) hypothesis. The TME is an entity containing different type of cells, undergoing a dynamic evolution through the intra crosstalk of these components and their interaction with malignant cells [107,108]. The development of sophisticated techniques to dissect the cellular and molecular composition of the TME led to gain knowledge of its role in tumor progression and in the responsiveness of cancer patients to immune-based therapy [107,108,109,110,111]. In addition, these studies contributed to define the hallmarks of cancer immune surveillance and evasion from immune responses [112,113,114] (Figure 1), which are associated with the mechanisms that drive the low immunogenicity in tumor cells and actively induce the immune suppression (Table 1) [115]. In this context, CSCs/CICs, due to their immunological privileged phenotype, can represent one of the components responsible for tumor dormancy [55]. The role of CSCs/CICs in the crosstalk with immune cells, although still not conclusive, has been described and is briefly summarized below (see also Table 1). 

The **natural killer (NK) cells** are members of innate lymphocytes which contain cytotoxic and proteolytic enzymes such as perforin, granzyme and granulysin [136]. NK cells are found to be efficient in recognizing and eliminating in vitro CSCs/CICs from various cancer types [84,85,116]. However, these mechanisms can occur only upon efficient expression by CSCs/CICs of target molecules for NK receptors and sufficient levels of NK cell activation [84,117,118,119]. The activatory receptors on NK cells are engaged upon the binding of either NKG2D to its ligands on target cells, such as stress-inducible MHC-like molecules MICA/B and ULBPs or the interaction of natural cytotoxicity receptors (NCR, NKp30 and NKp44) with their specific ligands. Studies on CSCs/CICs isolated from CRC, GBM and melanoma highlighted that NK cell-mediated killing of CSCs/CICs can occur only when they express low levels of inhibitory HLA class I molecules and detectable/high levels of NKG2D ligands [61,84,85,116,119]. In another study, stem cells from acute myeloid leukemia (AML) showed low expression of the ligands of NKG2D MICA/B and ULBPs [120]. Therefore, the levels of expression of target molecules for either the activating receptors of NK cells or HLA molecules interacting with killer-cell immunoglobulin-like receptors (KIRs) [121] represent mechanisms to tune the susceptibility of CSCs/CICs to NK cells [86].

The **dendritic cells (DCs)** belong to the professional antigen presenting cell (APC) subtype that can induce both innate and adaptive immune responses [137,138]. They are important in the antigen presentation and activation of T and B lymphocytes [139]. iDCs can generate the regulatory T lymphocytes (Tregs) or Tr-1 cells with suppressor activity [139,140,141]. iDCs undergo differentiation to form the mature DC and they elicit stimulatory signals to T cells. However, within the TME their differentiation can be affected by the immunosuppressive environment resulting in the impairment of effector T-cell mediated responses [122]. DCs can also promote the survival and proliferation of CSCs/CICs through the chemokine (C-X-C motif) ligand 1 (CXCL1) signaling [123]. This pro-tumoral activity was inhibited by the treatment with the CXCL12/CXCR4 inhibitor, AMD3100 [124].

CSCs/CICs from AML showed superior level of expression of CD47 as compared to normal hematopoietic stem cells [125]. CD47 is the ligand of the signal regulatory protein alpha (SIRPα) that is involved in the regulation of the phagocytosis of macrophages and DCs. CSCs/CICs from AML, through the engagement of CD47, can inhibit the cell-mediated phagocytosis, resulting in the impairment of innate immune responses [125].

**Macrophages** are important players of the innate responses; these cells are endowed with phagocytosis functions and can migrate to tissues, where their differentiation takes place [142]. Macrophages present at the tumor tissues are named tumor-associated macrophages (TAMs). These cells can either display positive or negative role in tumor’s prognosis and, in the context of TME, can polarize toward M1 and M2 subtypes that exert anti-tumoral or protumoral activity, respectively [126,142]. The polarization of M2 macrophages is mediated by either immunoregulatory or pro-inflammatory cytokines such IL-4 and IL-10 or IL-6, respectively, chemokines CCL2 and CXCL12 and TGF-β [142]. This immunosuppressive microenvironment generated by TAMs can result in the impairment of T-cell activation and proliferation [126,143]. TAM can also promote stemness functions of cancer cells through the STAT3 signaling pathways [127,128,129]. The activation of STAT3 pathway is central for the bidirectional crosstalk between TAM and CSCs/CICs [130].

The **myeloid derived suppressor cells (MDSCs)** are heterogeneous populations of immature myeloid cells endowed with the ability to suppress the effector functions of immune responses [143,144,145]. MDSCs can inhibit the anti-tumor activity of DCs, T, NK and NKT cells [131]. Thus, MDSCs promote the maintenance of CSCs/CICs. The activation of STAT3 is one of the important mechanisms required for the CSCs/CICs survival and proliferation [128,129]. STAT3, which is aberrantly expressed in CSCs/CICs, can induce the differentiation of monocytes towards MDSCs that can orchestrate the EMT and the generation of CSCs/CICs in solid tumors [132]. In addition, STAT3 is crucial in the crosstalk between CSCs/CICs and their niche [131]. MDSCs and TAM within the tumor ecosystem of patients with leukemia have been shown, on the one hand, to sustain and promote the survival and proliferation of CSCs/CICs and, on the other hand, to impair T cell-mediated responses [133,134].

The **regulatory T cells (Tregs)** are subset of CD4^+^ T cells that act as physiological regulation of innate and adaptive immunity through the expression of immune checkpoints and the secretion of immunoregulatory cytokines [126,131]. Additionally, the immunosuppressive TME, through soluble factors such as IL-10, IL-13, TGF-β and Galectin 3 can lead to the differentiation of CD4^+^ T cells into Tregs, which can inhibit effectors functions of T cells and the activity of APCs [2,56,126].

**B cells with regulatory functions (Bregs)** are associated with immunosuppressive TME and poor prognosis when present in tumor lesions of solid tumors [126,135]. These cells can promote, through the secretion of cytokines like IL-6, IL-10 and TGF-β, the differentiation and expansion of TAMs, MDSCs and Tregs [126,135]. In addition, Bregs play an immune evading role by inducing the expression of the immune checkpoint cytotoxic T lymphocyte antigen-4 (CTLA-4) and suppressing the proliferation of CD4^+^ T cells [135]. Their role in regulating CSCs/CICs is not fully established; however, they might contribute, at least in an indirect manner, to establish the niche and to its crosstalk with cells with stemness properties.

The complexity of the TME and the lack of in vivo model to monitor the crosstalk of CSCs/CICs with the niche and immune cells prevent to achieve conclusive evidence regarding the mechanisms undergoing the regulations of CSCs properties by the immune system and vice versa. Further studies are required to elucidate this important topic. These findings will allow tailoring immune interventions aimed at skewing the immunosuppressive TME toward anti-tumor functions.

## 4. Role of IDO in Immunomodulation

**Figure 2:**
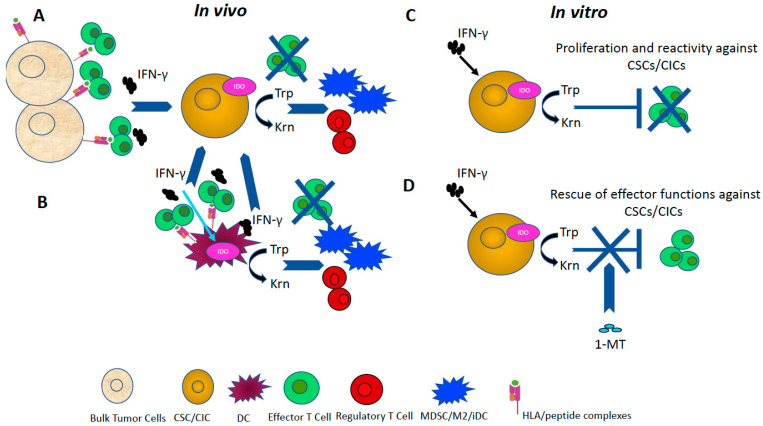
IDO is a key regulator of immunoregulatory properties of CSCs/CICs. IDO is a key regulator of anti-tumor immune responses. In an in vivo model (Panels (**A**,**B**), the expression of IDO in either tumor cells (Panel (**A**)) or APC (Panel (**B**)) can be induced by IFN-γ. IFN-γ can be released in the TME by effector cells activated through the engagement of TCR by HLA/peptide complexes expressed by either differentiated tumor cells (Panel (**A**)) or APC (Panel (**B**)). IFN-γ can then upregulate IDO in either CSCs/CICs or APC, leading to tryptophan degradation into kynurenine and its deprivation in the TME. Then, the inhibition of effector T cells and the differentiation of Tregs, MDSCs, M2 and iDCs occur, resulting in the impairment of efficient anti-CSC/CIC immune responses (Panels (**A**) and (**B**), respectively). The production of IDO by APC can also be mediated by the IFN-γ in either autocrine manner or when the cytokine is exogenously provided by components of the TME (Panel (**B**)). The in vitro model can be utilized to assess the IFN-γ induced expression of IDO by CSCs/CICs and its role in inhibiting both the proliferation and the anti-CSC/CIC reactivity of T effector cells (Panel (**C**)). T cell-mediated immune responses against CSCs/CICs can be rescued by neutralizing the activity of IDO with the specific inhibitor 1-MT (Panel (**D**)). CSC/CIC, cancer stem cell/cancer initiating cell; HLA, human leukocyte antigen; iDC: immature and tolerogenic dendritic cell; IDO, indoleamine 2,3- dioxygenase; IFN, interferon; MDSC, myeloid derived suppressor cell; M2 macrophages: immunomodulatory/suppressive macrophages; TME, tumor microenvironment; Treg, T regulatory cells; 1-MT, 1- Methyl Tryptophan.

IDO, a heme-containing oxidoreductase, is involved in the catabolism of tryptophan, which is essential in many types of cells, including T cells, DCs, macrophages and fibroblasts [68,146]. Two types of IDO, IDO1 and IDO2, are involved in the degradation of tryptophan with different rates [147]. IDO is seen to be overexpressed in most human tumors [148]. IDO has been identified as a critical factor involved in aiding immunosuppressive functions in the TME and it can be upregulated by IFN-γ (Figure 2) [55,70]. A relationship between the attenuation of Bin-1, a cancer suppressive gene, and IFN-γ mediated IDO expression has been described [149].

IDO plays important role in carcinogenesis and tumor progression, and its expression was detected in different types of solid tumors, such as CRC, prostate cancer, breast cancer, GBM and melanoma. The expression of IDO was also found to be associated with the prognosis of tumors [68,150,151,152,153].

IDO induces immune tolerance by the negative regulation of natural killers (NK) and T cells and the induction of MDSCs (Figure 2) [153]. This enzyme can inhibit the differentiation and proliferation of effector T cells and induce the generation of Tregs (Figure 2) [55,70,154,155].

IDO can play additional and distinct functions by regulating the differentiation of plasmacytoid DCs (pDCs) [156]. Through the induction of IDO, TGF-β can skew pDCs from immunogenic to tolerogenic cells, although the molecular pathways linking TGF-β to IDO have not been fully elucidated yet [156,157].

IFN-γ and TGF-β1 can both orchestrate IDO through different mechanisms. pDCs that are IFN-γ-conditioned suppress the proliferation and induce the apoptosis of T cells [156]. TGF-β-conditioned pDCs preferentially induce Treg cells that express the transcription factor Foxp3 [157]. The inhibitor of IDO, 1-methyltryptophan (1-MT), can counteract the inhibition of proliferation and apoptosis of T cells mediated by IFN-γ while it has no effect on the TGF-β-mediated activity on pDCs [156,157]. IDO can exert double functions. On the one hand, it catalyzes tryptophan depriving the T cells of important nutrients. On the other hand, IDO can also act as a signal transducer upregulating the non-canonical NF-κB pathway and, through the increased levels of TGF-β1, leading to long-term immune tolerance [156,157]. Moreover, IDO can be induced in DCs by the engagement of the immune checkpoint molecule CTLA-4 by CD80 and CD86 expressed on APC [158].

IDO was also detected in tumor initiating cells isolated from different types of tumors (GBM, breast and pancreatic cancer) [159]. The expression of IDO could be blocked by the specific inhibitor mitoves and transcriptional and post-transcriptional mechanisms regulating the expression of IDO [159]. IDO was found to be overexpressed in CSCs/CICs vs. bulk tumor cells isolated from either GBM or CRC upon pre-treatment of the cells in vitro with IFN-γ [160] (igure 2). IDO was found as one of the agents responsible for the inhibition of the generation in vitro of T-cell mediated responses against CSC/CICs, as both their proliferation and anti-tumor reactivity could be rescued in the presence of the IDO inhibitor 1-Methyl Tryptophane (1-MT) [160] (Figure 2). IDO has been shown to regulate also the tumor progression, invasion and metastasis through the crosstalk of CSCs/CICs and stromal cells in the TME [70]. The inhibition of IDO can block the CSC/CIC-mediated invasiveness and progression of tumors [70] (Figure 2).

IDO is a pivotal molecule for the impairment of T cell-mediated responses. Although this aspect is not fully elucidated yet, the blockage of the IDO pathway could be utilized as an effective method for neutralizing the immunomodulatory effects of CSCs/CICs. Further investigations should be undertaken for the development of a therapeutic strategy that can result in eliciting patients’ immune responses and in tumor eradication [152,161]. The evaluation of the clinical efficacy in metastatic melanoma patients of the inhibition of IDO in combination with the antagonistic antibody targeting the immunoregulatory molecule programmed cell death-1 (PD-1) resulted in high grade of toxicity and the anticipated closure of the Phase III clinical study, indicating that further investigations on the mechanisms of action of IDO inhibitors and their synergy with other therapeutic interventions need to be performed. Nevertheless, multiple Phase I/II clinical studies are currently evaluating the clinical efficacy of the IDO inhibitor Epacadostat (INCB024360) in association with standard therapy, targeted therapy, immunotherapy (cancer vaccines or immune checkpoint blockade) or the combination of these approaches for patients with different types of solid tumors or lymphoma refractory to previous treatments (NCT01961115, NCT03823131, NCT03589651, NCT03532295, NCT03471286, NCT03006302, NCT04463771, NCT03328026 and NCT03322384).

## 5. Immunomodulatory Factors Associated with CSCs/CICs

The immunomodulatory activity of CSCs/CICs also occurs through the secretion of a variety of soluble factors and/or the expression on the cell membrane of immune checkpoints (Table 2).

### 5.1. Cytokines, Growth Factors and Other Soluble Regulators

IL-4, IL-13 and IL-10, which are either immunoregulatory or pro-inflammatory cytokines, have been described to be released by CSCs/CICs from different type of solid tumors, leading to the impairment of T helper type 1 (TH1)/effector immune responses [52,55,56,62,65,162]. IL-10 secreted by CSCs/CICs can inhibit the anti-tumor activity of CD4^+^ T cells and drive their differentiation toward Tregs [55,162]. IL-4 autocrine signaling on CSCs/CICs isolated from CRC led to TH2 type differentiation of CD4^+^ T cells and the failure in eliciting antigen-specific T cell responses upon the co-culture with autologous peripheral blood-derived T cells [80]. The neutralization of IL-4-signaling with a specific mAb was able to restore tumor-specific T cell responses and their ability to target CSCs/CICs [80]. In the context of the same in vitro model, the pre-treatment of CRC-CSCs/CICs with immunomodulatory agents (IFN-γ) could increase the antigen processing and presentation of these cells and induce antigen-specific immune responses against a neoantigen generated by a non-synonymous mutation detected both in tumor cells with stemness properties and in the bulk tumor cells [103].

Interestingly, macrophages and MDSCs through the production of IL-13 and IL-4 [163] can drive the EMT and the development of CSCs/CICs [164]. Along this line, IL-6, IL-8, IL-10 and IL-13 (Table 2) can play an important role in the maintenance of an immune suppressive TME and can regulate the interaction of CSCs/CICs with the niche [52,165].

Galectin-3, a carbohydrate binding protein, has been reported to be expressed by CSCs/CICs as well as, through the interaction with proteins both at the extra- and intra-cellular levels, to regulate diverse signaling pathways, including EMT and cancer stemness [170,171,172,173]. Galectin has been also shown to orchestrate immunoregulatory functions in CSCs/CICs and to mediate negative regulatory functions in the TME [133,174,175].

The growth differentiation factor-15 (GDF-15) is a member of the TGF-β superfamily that promotes the immune escape by inhibiting the expression of MHC class II and co-stimulatory molecules on DCs and increasing the levels of TGF-β1, which prevents the maturation of DCs [167,168]. Similarly, it can mediate the differentiation of macrophages into tolerogenic subtype [167]. The final outcome is the inhibition of T cell activation and differentiation into effector cells [167]. Although the knowledge of the mechanisms of GDF-15 in shaping the TME toward the immune evasion and a pro-tumoral ecosystem is still at the infancy, the involvement of few pathways, such as TGF-β, PI3K/mTOR and MAP kinases, has been determined [134,167,168].

Leukemic-CSCs/CICs can also lead to the differentiation of MDSCs and TAM and the consequent impairment of T cells by the overexpression of T cell immunoglobulin mucin-3 (TIM-3)/Galectin 9 pathway [133,134]. The same signaling pathway can also sustain the survival of leukemia stem cells [133].

Prostaglandin E_2_ (PGE2) was detected in several types of malignancies, with frequent association with poor prognosis. For instance, in vivo experiments in a model of uterine cervical cancer showed that MDSCs can secrete PGE2 and promote the stemness properties of malignant cells [169].

### 5.2. Immune Checkpoints

The PD-1/programmed cell death ligand 1 (PD-L1) axes and the Cytotoxic T Lymphocyte Antigen-4 (CTLA-4) represent immune checkpoints molecules involved in the negative regulation of cell-mediated immune responses. These molecules mediate the physiological regulation of immune responses to prevent the development of autoimmune reactions, however their expression has been found to be upregulated in cancer patients [182]. The development of clinical grade blockade agents that can unleash the anti-tumor immune responses opened a new scenario for the therapeutic interventions of cancer patients, even at the advanced stages of the disease [183,184]. The advances in the clinical usage of immune checkpoint blockade and the identifications of biomarkers associated with patients’ responsiveness to these therapies allowed these interventions to become the standard of care for different type of tumors. However, some patients are unresponsive or develop resistance to these therapies [184]. Therefore, many studies are ongoing to evaluate the clinical efficacy of the combination of immune checkpoint blockade with either standard therapy, or other immune-based approaches [184,185].

PD-L1 and CTLA-4 were detected in CSCs/CICs with the ability to inhibit the survival and induce the exhaustion of antigen-specific T cells, as well as leading to the differentiation of immunosuppressive subsets of immune cells [55,62,64]. It has been shown that the high levels of PD-L1 in CSCs/CICs is due to EMT and to EMT/β-catenin/STT3/PD-L1 signaling axis [177,178]. Moreover, PD-L1 expression can be stimulated by PI3K/AKT and RAS/MAPK pathways [177,178]. As a consequence of PD-L1 overexpression in CSCs/CICs, these cells gain not only the ability to escape the immunosurveillance but also to hinder the anti-tumor activity of T and NK cells. Therefore, the antagonistic mAbs targeting the immune checkpoints could represent relevant tools to be utilized also in combination with other approaches to unleash the immune system to target CSCs/CICs.

Another molecule essential in the immune regulation is B7-H3. The B7 protein family plays a critical role in the regulation of the responses of activated T cells. The B7 superfamily consists of eight members, including B7-H3. All B7 superfamily members are transmembrane proteins that comprises both extracellular and intracellular domains along with transmembrane domain. These molecules exert both inhibitory and stimulatory effects on T cell activation [41]. B7-H3 is encoded by the *CD276* gene, and it was first identified as an immune molecule expressed in antigen-presenting cells or macrophages. It acts as a second signal molecule with regulatory functions for T cells. Recent reports showed that B7-H3/B7-H4 promotes cancer metastasis in patients, including solid tumors, such as CRC, prostate, pancreatic and brain tumors [179,180,181].

The STAT3 pathway is involved in the maintenance of stemness properties in tumor cells and sustaining their proliferation [128,129]. This molecular pathway regulates multiple process occurring in the TME for the crosstalk between CSCs/CICs and TAMs and, as secondary effect, mediates the impairment of T cell immune responses [130,145]. STAT3 activation can induce monocytes to acquire MDSC properties in pancreatic cancer tissues [186]. On the other hand, MDSCs can induce stemness features in tumor cells and induce EMT in pancreatic adenocarcinoma [186].

The molecular pathways that are aberrantly expressed in CSCs/CICs lead to the downstream induction of molecules endowed with immunoregulatory functions that can orchestrate the crosstalk between TME and the immune cells toward immune suppressive and pro-tumoral subtypes (Table 2). These molecular signaling can also promote and/or sustain the stemness features of tumor cells. Further investigations are warranted to understand the mechanisms regulating the immunoregulatory profile of CSCs/CICs and their relationship with the TME. The obtained results would contribute to the identification of possible therapeutic interventions that can revert the immunogenic profile of cancer cells endowed with stemness properties and will allow to understand the immune-related mechanisms regulating the generation and maintenance of CSCs/CICs and of their niche. Nevertheless, deep analyses are required to build the complex interactions of molecules and signaling pathways and identify the make-up of cells that can orchestrate the immune surveillance in the TME. In addition, due to the multiple immunomodulatory molecules (Table 2) expressed by CSCs/CICs and their mutual connection, studies to prioritize their inhibitions in the context of therapeutic interventions or of combinations of different approaches are warranted.

## 6. The Dual Role of microRNAs in Orchestrating Pro-Tumoral and Stemness Features and Immunological Functions

MircroRNAs (miRNAs) are a new class of non-coding RNAs that work by binding to a target mRNA in order to regulate at post-transcriptional level the gene expression [71]. The initial discovery was in *Caenorhabditis elegans* which indicated the vital role of miRNA in regulating the molecular mechanisms associated with embryogenesis and mammalian stem cells. The depletion of the enzyme DICER, which regulates the biosynthesis of miRNAs, led to lethality in mouse due to the inhibition of embryonic stem cells [187,188]. Studies have shown that the aberrant expression of miRNAs is linked to human malignancies by promoting anti-apoptotic activity, proliferation, invasion and formation of metastasis by malignant cells [189]. Single miRNAs are able to regulate the expression of multiple genes. Despite the complexity of functions, they represent a potential strategy for targeting CSCs/CICs [190,191].

### 6.1. MIRNAs Regulating Tumor and Stemness Properties

CSCs/CICs have been reported as enriched in miRNAs with oncogenic features (Table 3) [192]. Oncogenic miRNAs, including miR-9, miR-181 and miR-215 have been linked to tumor onset and resistance to therapy caused by CSCs/CICS, while let-7, miR-16, miR-34, miR-122, miR-152 and miR-218 have been described as tumor suppressors and they have been reported to be lost in CSCs/CICs [192,193,194].

MiRNAs exhibit dual functions and some of them might act as both tumorigenic and tumor suppressor. For instance, upregulation of miR-34 and let-7 promotes tumor suppression, while their down modulation leads to oncogenesis [193,217]. The link between miRNAs and CSCs/CICs has been proven by recent studies showing the ability of miRNAs to regulate CSCs/CICs characteristics, such as self-renewal and drug resistance [246]. For example, miR-29a is upregulated in BCSCs, leading to their migration and metastasis as a result of the induced basic fibroblast growth factor (bFGF) [212]. In CRC, miR-17-92 has been found to exert an anti-apoptotic effect leading to the development and progression of CRC carcinogenesis [76]. In CRC stem cells, the overexpression of miR-27a has been reported and its knock down led to the susceptibility of CRC stem cells to immune cells and the activation of the apoptotic pathways [208].

MiR-21 is of particular interest as it is a well-studied “oncomiR”. It has been reported as linked to a poorer outcome when over expressed in hepatocellular carcinoma, renal cell carcinoma, pancreatic, lung adenocarcinoma, low-grade glioma and GBM [202]. In a recent study by our group, miR-21 expression resulted significantly lower in CSCs/CICs as compared to their differentiated counterparts in GBM [160]. MiR-21 represents a key regulator of the EMT signaling that has been shown to be one of the earlier players in CSC/CIC formation [203]. MiR-21 has also been reported in another study to be down-regulated in GBM CSCs/CICs as compared to astrocytes [204] and shown to be associated with a differentiated phenotype in GBM [205,247].

Other miRNAs have been shown to be dysregulated in CSCs/CICs. The low expression of MiR-200 and let-7 in CSCs/CICS affects EMT stem-like transition, self-renewal and metastasis in multiple tumors, including breast and prostate cancer and CRC while the lower expression of miR-34a in CSCs/CICS affects self-renewal and asymmetric division [238,248,249,250,251]. BCSCs can release microRNAs belonging to the MiR-200 family in association with the ability to form metastases [252]. In GBM, the downregulation of miR-10b inhibited CSC/CIC proliferation and metastasis [195].

### 6.2. MiRNAs with Immunological Roles

MiRNAs are also involved in regulating and orchestrating the immunological functions, and it is unsurprising that irregularities in their expression can lead to an altered tumor immune surveillance that contribute to cancer initiation and progression (Table 3) [75]. Multiple miRNAs have been associated with the regulation of innate immune functions [224]. For instance, miR-155, miR-125a/b, miR146a, miR-21 and let-7 play a key role in the differentiation and activation of macrophages and DCs, through toll like receptors (TLRs) and IFN-β [209,224]. DCs deficient for the expression of miR-155 display defective antigen presentation and are not able to elicit antigen-specific immune responses [225]. miR-150, miR-155 and miR-181 are regulators of the differentiation of NK cells which are key elements of the innate immune response [72,76,209]. MiRNAs are also important players in the orchestration of T cell-mediated responses [77,224,253]. The differentiation and the tuning of antigen specificity by the TCR of T lymphocytes is mediated by miR-181 [236,237]. T helper effector is also governed by miR-155 that, together with miR-142 and miR-146a, can also regulate the differentiation and functions of T_reg_ cells [84].

Therefore, miRNAs can also represent key molecular regulators of pathways involved in the immunological profile of tumor cells, including CSCs/CICs. MiR-34a/c and MiR-10b are involved in the regulatory process of the expression of NKG2D ligands (MICB and ULBPs) on tumor cells [196,218], the expression of which on CSCs/CICs, as described in Section 1, allow the efficient recognition by NK and T cells through stimulation and co-stimulation, respectively [55,56]. Interestingly, miR-17-92 cluster, particularly miR-20a, can negatively regulate the expression of the NKG2D ligands ULBP2 and MICA/B in BCSCs and influence the susceptibility of these cells to NK cells [86]. This phenomenon occurred in breast cancer through the inhibition of the MAPK/ERK signaling pathway.

The silencing of this miRNAs can drive the efficient recognition in vitro of these cells by NK cells and rescue the immune escape in vivo, through the upregulation of NKG2DLs [199]. These mechanisms are regulated epigenetically, since the treatment of the cells with histone deacetylase inhibitors (HDACis) can inhibit the expression of miR-17-92 cluster and restore the susceptibility of tumor cells to NK immune responses [199]. Thus, targeting of miRNAs with antisense inhibitors or HDAC may represent a novel approach for increasing the immunogenicity of BCSC.

Along this line, miRNAs associated with the expression of immune checkpoints have been also described, e.g. miR-200 and miRNA-34 can regulate the expression of PD-L1 [206,219]. In other cases, the overexpression of positive regulators (e.g., miR-24 and miR-181) of PD-L1 was detected in different type of solid tumors [206]. Some of these miRNAs (e.g., miR-21 and miR-181) modulate the expression of PD-L1 through the regulation of two pathways: PTEN and STAT3 [206].

The expression of PD-L1 has been reported also regulated by additional miRNAs. For example, miR-142-5p has been shown to inhibit PD-L1 expression by binding to its 3′UTR in pancreatic tumor [221]. Another important player in the inhibition of PD-L1 worth of note is represented by miR-155. The expression of miR-155 through the exposure of cells to TNF-α and IFN-γ suppressed the expression of PD-L1 in lymphatic endothelial cells and fibroblasts [226]. MiR-155 also activated STAT3 signaling and promoted tumor progression in breast cancer [227,228]. The inhibition of immune checkpoints can also be indirect. As an example, a low expression of PTEN induces the upregulation of PD-L1 in cancer cells. PTEN is downregulated by miR-20b, miR-21 and miR-130b while enhancing the expression of PD-L1 [200]. Another miRNA known to regulate PTEN expression is miR-214a, which has been reported to control the expansion of T cells in solid tumors [254]. MiR-21 and miR-181-1b downregulate PTEN through STAT3 in CRC [207]. MiR-21 also controls the polarization of macrophages through restraining the IFN-γ induced STAT1 signaling [202]. In addition, the upregulation of miR-21 expression has been reported to suppress lymphocyte migration via the inhibition of activated STAT3 signaling pathway [202,207]. The JAK/STAT pathway also intervenes in the modulation of macrophage polarization through miR-23, miR-24-2 and miR-27a [210].

The axis of miR-155 and miR-143, which is regulated through the control of TGF-β1 by SMD3 and SMT4, leads to upregulation of B7-H3 and B7-H4 in CRC [229]. In glioma, miR-29 and the mutational status of the isocitrate dehydrogenase (IDH) can influence the expression of B7-H3, with some variabilities depending on the grade of the tumors [213]. The same miRNA has been found to be a key regulator of B7-H3 in melanoma patients whose levels are inversely correlated with the expression of the target molecules and with the progression of the disease [214]. B7-H3, besides the role of immune checkpoint in immune responses, can promote proliferation and invasiveness of tumor cells [213,214]. Along this line, the downregulation of miR-214 induced the expression of B7-H3, including its soluble forms, and the subsequent polarization of macrophages to the M2 subset, which is mediated by the JAK2/STAT3 pathways [240].

MiR-448 was reported as a negative regulator of IDO and of the related differentiation of Tregs in breast cancer, while miR-142 played an important role in promoting an immunosuppressive TME through the induction of IFN-γ, leading to the expression of IDO in lymphatic endothelial cells [222,242]. Post-transcriptional regulation of IDO has been described for miR-181, miR-155 and miR-760 [159,255].

Interestingly, a study performed by our group identified a differential profile of miRNAs in CSCs/CICs and autologous bulk tumor cells isolated from GBM [160]. These miRNAs included the aforementioned regulators of immunological pathways, such as TGF-β, STAT3, IFN-γ, T signaling and differentiation [160]. Furthermore, this pattern of miRNAs was associated with the differential expression of some immune related genes and, in some cases, proteins observed in CSCs/CICs vs. bulk tumor cells [61].

Of note, dysfunctions in the immune-related molecules described above occur in CSCs/CICs, leading the TME/niche toward a pro-tumoral ecosystem and determining the low susceptibility of these cells to immune responses and their immune evasion (Table 3). The targeting of miRNAs with immune-modulatory functions that have been shown to be aberrantly expressed in CSCs/CICs represents a powerful strategy to foster antitumor immunity and obtain a better response in cancers that are resistant to treatment. Therefore, investigations of the role of miRNAs in the light of cancer stemness properties and of their crosstalk with the TME and the immune system are encouraged to either develop new tools or optimize the targeting of these cells with immunotherapy.

## 7. Conclusions

The investigations conducted on CSCs/CICs to characterize the molecular and immunological properties of cancer patients have shed light on the various molecules and signaling pathways conferring the properties of CSCs/CICs. Moreover, heterogeneity and plasticity of these cells contribute to the complexity of the malignancies and the difficulties in isolating CSCs/CICs with a unique phenotype. These cells have a constant interplay with the TME/niche that determines the fate and plasticity of these cells. CSCs/CICs and the TME represents a dynamic ecosystem in continuous evolution. The lack of unique markers associated with CSCs/CICs and in vivo models to monitor the interactions of these cells with the TME and the immune system prevent fully understanding of the mechanisms that promote the stemness features of malignant cells and the tumor dormancy.

Along with the resistance of CSCs/CICs to standard therapy, they display low immunogenicity and multiple mechanisms leading to low/absent susceptibility to the immune responses and immune evasion (Figure 1). The down modulation of HLA molecules, APM components, NKG2D ligands and TAAs can lead to the impairment of cell-mediated immune responses. However, the mechanisms regulating this phenomenon are still unknown. A systematic analysis of these features should be performed on CSCs/CICs isolated from multiple type of tumors. Various immunomodulating molecules can either inhibit the immune surveillance against tumors or skew the immune responses toward immune suppressive environment (Table 2). Immune checkpoint blockade drugs have been widely utilized for the therapeutic treatment of cancer patients and represent in some cases standard of therapy for aggressive tumors with limited options of interventions or that develop resistance to other line of treatments [256,257,258,259]. Therefore, these agents could be feasible to revert the immunosuppressive profile of CSCs/CICs, however the multiplicity of molecules involved in the phenotype of these cells limits the efficacy of a single drug in targeting these cells. The optimal combinations of immune-based therapeutic interventions are still under debate and several clinical trials are ongoing to achieve this knowledge. Nevertheless, the evidence that the clinical efficacy of the interventions that have been previously listed is limited to subgroups of patients [55,57], and the development of resistance by patients on the course of treatment might be explained by the failure of these drugs in targeting efficiently CSCs/CICs and in reverting their low susceptibility to immune responses.

MiRNAs represent key regulators of the principal functions of tumorigenicity, stemness and immune functions. They represent a complex network that orchestrates the above functions and, therefore, are also relevant for the mechanisms conferring stemness features (Table 3). MiRNA-based therapeutic approaches are based on the blockade of miRNA with oncogenic features (through anti-miRNA constructs, miRNA sponges or masks and small molecule inhibitors) and enhancing the expression of miRNAs with tumor suppressing features (through synthetic miRNA precursors or miRNA mimics) [250]. Pre-clinical studies in vitro and in vivo have been conducted with some success. Few types of miRNAs have also been utilized in Phase I/II clinical trials, although some toxicities have been registered [206]. However, these approaches still need to be optimized and implemented for the efficient and specific targeting of cancer cells in vivo. In particular, the peculiar dual role of miRNAs and their capacity of targeting multiple genes warrant accurate and strong pre-clinical studies to optimize the specificity of the interventions and to prevent unwanted in vivo toxicities.

The role of miRNAs as tumor suppressor or oncogenic mediators needs to be well considered in the light of the development and progression of cancer. In addition, the same miRNAs, depending on its expression in different types of cells, such as immune cells or tumor cells, can both elicit anti-tumor immune responses and promote tumor growth. Examples are represented by the “oncomiR” miR-21 and miR-155. Therefore, to exploit miRNAs as therapeutic tools the correct cellular context and the accurate choice of mimicry or inhibition should be extensively investigated.

The understanding of miRNA regulatory role in tumor-mediated immunity and their association with CSCs/CICs will ease the developing of miRNA-based immunotherapy that can lead to the efficient eradication of the component of tumor resistant to therapies.

**Figure 3:**
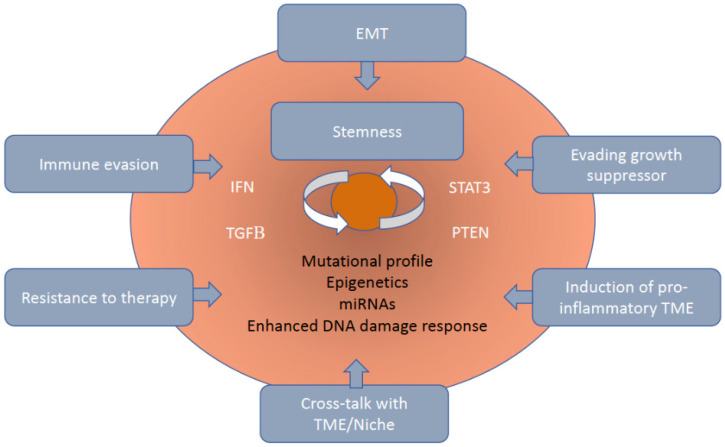
The hallmarks of immune escape of CSCs/CICs. The immune privileged phenotype of CSCs/CICs is the result of aberrant genomic, epigenetic and post-transcriptional processes. Moreover, the interaction of multiple signaling pathways, either up-regulated or down-regulated, and the dynamic cross-talk between cancer cells and the TME drive the induction and maintenance of stemness functions, as well as the generation of immunosuppressive environment and immune evasion. Inside the CSC/CIC (in orange) are indicated the principal genomic, epigenetic and post-transcriptional mechanisms regulating the phenotypic make-up of CSCS/CICs. The major signaling pathways involved in the crosstalk between CSCs/CICs and TME are indicated in white inside the cell. The grey rounded rectangles represent the major processes regulating the interplay between stemness and TME/immune system. CIC, cancer initiating cell; CSC, cancer stem cell; EMT, epithelial-to-mesenchymal transition; IFN, interferon; PTEN, phosphatase and tensin homolog; STAT3, signal transducer and activator of transcription 3; TGF-β, transforming growth factor beta; TME, tumor microenvironment.

The hallmarks of the immune evasion of CSCs/CICs have been identified (Figure 3), although the comprehensive knowledge of the underlying mechanisms still needs to be achieved. Immunotherapy represents the fifth pillar of the therapeutic interventions available for cancer patients (the different immunotherapy strategies are discussed in other publications [2,55]). Nevertheless, the usage of tools to modulate the activity of miRNAs represents an appealing approach to improve the clinical efficacy of immunotherapy, including combinations with other interventions. The achievement of insights of miRNAs regulating stemness properties and their crosstalk with the TME will contribute to understand the mechanisms regulating the tumor development, immune evasion and resistance to therapies.

## Figures and Tables

**Table 1:** The crosstalk between immune cells and CSCs/CICs.

Immune Responses	Cell Type	Effect of CSCs/CICs	References
Innate			
	NK cells	Immune evasion of CSCs/CICs or their killing depending on the expression profile of HLA molecules and ligands of NK activatory receptors.	[84,85,86,116,117,118,119,120,121]
	DCs	1. Differentiation towards iDCs and impairment of anti-tumor (including CSCs/CICs) T cell-mediated responses. 2. Pro-tumoral role by CXCL1 and CXCL12/CXCR4.	[122,123,124,125]
	Macrophages	Promote stemness functions through STAT3 signaling pathway.	[126,127,128,129,130]
	MDSCs	1. Promote stemness functions and survival of CSCs/CICs through STAT3 signaling pathway. 2. Impairment of anti-tumor (including CSCs/CICs) immune responses.	[131,132,133,134]
Adaptive			
	T lymphocytes	Immune evasion of CSCs/CICs or their killing depending on the levels of the expression of HLA molecules, APM and ligands of T cell co-stimulatory receptors.	[53,55,79,80]
	Tregs	Differentiation of Tregs through either the secretion of immunosuppressive factors by CSCs/CICs or their crosstalk with immune suppressive APC (e.g., iDCs, MDSCs, TAMs).	[55,79,126]
	Bregs	Establishment of the niche and sustainment of stemness functions.	[126,135]

APM, antigen processing machinery; Bregs, B regulatory cells; CSCs/CICs, cancer stem cells/cancer initiating cells; CXCL1 and CXCL12, chemokine (C-X-C motif) ligand 1 and 12; TAM, tumor associated macrophages; CXCR4, CXC-chemokine receptor type 4; DC, dendritic cells; iDC, immature and tolerogenic dendritic cells; MDSCs, myeloid derived suppressive cells; Tregs, T regulatory cells.

**Table 2:** Immunomodulatory molecules in tumors and CSCs/CICs.

Molecule	Cancer Types	Functions Mediated by CSCs/CICs	References
**Cytokines and growth factors**			
IL-4, IL-13	CRC, GBM, Ovarian, Lung, Breast, Pancreatic and Bladder cancer	Immunoregulatory functions: inhibition of effector functions; differentiation of immunosuppressive cells.	[52,65,79,80,162,163]
IL-10	Multiple	Immunoregulatory functions: inhibition of effector functions; differentiation of Tregs and MDSCs.	[163,164,165]
TGF-β	Melanoma, Pancreatic adenocarcinoma, GBM	Growth factor with both immunoregulatory functions and pro-tumoral activity.	[79,166]
GDF-15	Glioma, CRC, Prostate Cancer, Melanoma	Growth factor with immunoregulatory activity. Inhibition of effector immune functions.	[167,168]
**Soluble regulators**			
PGE2	Uterine Cervical Cancer	Lipid mediator of inflammatory and immune responses. Inhibition of innate immune responses.	[169]
Galectin-3	GBM, CRC, Ovarian and Breast cancer, Leukemia	Regulate the interaction of cells with TME. Inhibition of T cell-mediated responses.	[133,170,171,172,173,174,175,176]
IDO	GBM, CRC, Lung, Breast and Pancreatic Cancer	Enzyme regulating tryptophan catabolism: mediating the differentiation of Tregs, inhibitor of effector cell proliferation and cytotoxic activity.	[70,159]
**Immune checkpoints**			
PD-L1/CTLA-4	Multiple	Inhibitors of cell-mediated immune responses	[55,62,64,79,177,178]
B7-H3/B7-H4	CRC, Melanoma and Brain Tumors	Inhibitors of cell-mediated immune responses	[41,62,179,180,181]

CTLA-4, cytotoxic T lymphocyte antigen-4; IDO, indoleamine 2,3-dioxygenase; IL-4, -10, -13, Interleukin-4, -10, -13; GDF-15, growth differentiation factor-15; PD-L1, programmed cell death ligand-1; PGE2, prostaglandin E_2;_ CRC, colorectal cancer; GBM, glioblastoma; MDSCs, myeloid-derived suppressor cells; TGFb-1, tumor growth factor b-1; TME, tumor microenvironment; Tregs, T regulatory cells.

**Table 3:** Representative miRNAs in tumors, CSCs/CICs and immune functions.

miRNA	Tumor Type of CSCs/CICs	Type of miRNA	Functions Associated with Stemness Properties	Target Molecules of miRNAs Involved in Immunological Signaling *^∂^*	References
miR-10b	Breast cancer, brain tumors, squamous cell carcinoma	Oncogenic	Proliferation, migration and invasiveness	MICA/B, ULBPs	[195,196,197,198]
miR-20a	CRC, breast cancer	Oncogenic	Cell growth, migration, invasiveness, autophagy, PTEN, PI3K/AKT, MAPK/ERK	PTEN, MICA/B, ULBPs	[86,199,200,201]
miR-21	CRC, GBM and other solid tumors	Oncogenic	EMT, stemness by TGF-βR2, PTEN, STAT3 (miR-18 and miR-23) *	IFN-β, TLR (regulated also by miR-125 and let-7), PTEN, STAT3 (miR-18 and miR-23) *	[200,202,203,204,205,206,207]
miR-27	CRC	Oncogenic	Resistance to TNF-related apoptosis	Differentiation of Macrophage and MDSC	[208,209,210,211]
miR-29	Breast, Melanoma, Glioma	Oncogenic	Migration and metastasis due to the upregulation of bFGF	IFN-γ, B7-H3	[212,213,214,215,216]
miR-34	Breast, prostate and other solid tumors	Tumor suppressor	Stemness by targeting Wnt/β-catenin, suppresses CD44 which inhibits self-renewal and metastasis of CSCs	MICA/B, ULBPs (miR-10) *, PD-L1 (miR-24, miR-30b, miR-1385p, miR-425p and miR-940) *	[76,206,217,218,219,220]
miR142	Breast cancer	Tumor suppressor	Stemness, proliferation, apoptosis	TGF-β, IFN-γ, IDO, PD-L1	[221,222,223]
miR155	Breast and liver cancer	Oncogenic	Proliferation of BCSCs by activating STAT3	IFNs, TLR, TGFb1, IDO, B7-H3 and B7-H4, STAT3	[72,159,208,209,215,224,225,226,227,228,229,230,231,232,233,234,235]
miR-181	Multiple solid tumors	Tumor Suppressor	Drug resistance, PTEN, STAT3/AKT (miR-18 and miR-23) *	PD-L1 (miR-940 and miR-30b) *, PTEN, STAT3 (miR-18 and miR-23) *, IDO (miR-760) *	[72,76,159,207,236,237]
miR200	CRC, breast and prostate cancer	Tumor suppressor	EMT, invasiveness, chemoresistance	PD-L1	[206,219,238,239]
miR214	Ovarian cancer	Tumor suppressor	EMT, invasiveness and proliferation, JAK2/STAT3	B7-H3, JAK2/STAT3	[206,240,241]
miR-448	Brain tumors, breast and hepatic cancer	Tumor suppressor	EMT, TGF-β1	IDO, TGF-β1	[222,242,243,244,245]

*^**∂**^* These mechanisms are also involved in the differentiation of different subsets of T cells, including Tregs, macrophages, DC and MDSCs; * They are also regulated by others miRNAs. DC, dendritic cells; EMT, epithelial to mesenchymal transition; bFGF, basic fibroblast growth factor, MDSCs, myeloid-derived suppressor cells; PD-L1, programmed cell death ligand-1; PTEN, Phosphatase and TENsin homolog; STAT3, Signal transducer and activator of transcription 3; TGF-β1, Transforming growth factor beta 1, TLR, toll like receptor; Treg, T regulatory cells.

## Abbreviations

ABCA5, ATP-binding cassette, sub-family A (ABC1), member 5; AML, Acute myeloid leukemia; ALDH-1, aldehyde dehydrogenase-1; APC, antigen presenting cells; APM, antigen processing machinery; BCSCs, breast cancer stem cells; CEP55, centrosomal protein 55; CIC, cancer initiating cell; CRC, colorectal cancer; CSC, cancer stem cell; CT, cancer testis; CXCL1, the chemokine (C-X-C motif) ligand 1; CXCR4, CXC-chemokine receptor 4; DCs, dendritic cells; EMT, epithelial-to-mesenchymal transition; HLA, human leukocyte antigen; IDO, Indoleamine 2,3- dioxygenase; GBM, glioblastoma multiforme; GDF-15, growth differentiation factor-15; HER-2, epidermal growth factor receptor type 2; hTERT, human telomerase reverse transcriptase; KLF4, Kruppel-like factors 4; IDO, Indoleamine 2,3-dioxygenase; IFN, interferon; IL-4, Interleukin 4; IL-6, Interleukin 6; IL-10, Interleukin 10; IL-13, Interleukin 13; mAb, monoclonal antibody; LGR5, leucine-rich repeat-containing G-protein-coupled receptor 5; LMP2, -7 and -10, low-molecular-weight proteins; miRNAs/miR-, MicroRNAs; MDSC, myeloid derived suppressor cell; MICA/B, major histocompatibility complex class I chain A related; NK cells, natural killer cells; NKG2D, natural killer group 2 member D receptor; NSCLC, non-small cell lung cancer; OCT-3/4, octamer binding transcription factor; PD-1, programmed death-1; PD-L1, programmed death ligand-1; PI3K/AKT; PGE2, Prostaglandin E2; phosphatidylinositol 3-kinase/ Protein Kinase B; PTEN, phosphatase and tensin homolog; RCC, renal cell carcinoma; SOX-2, sex determining region-Y-box 2; STAT3, signal transducer and activator of transcription 3; TAA, tumor-associated antigen; ULBP, UL16 binding protein 1; VEGF, vascular endothelial growth factor; TME, tumor microenvironment TGFβ-1, transforming growth factor β-1; TH, T helper type 1; TH2, T helper type 2; TLR, toll like receptor; Treg, T regulatory cells; ULBP2, UL16 binding protein 2.
